# What Can Neurosurgical Pediatric Populations Do in Functional Magnetic Resonance Imaging? Brain Activity Mapping Before Intervention Tasks, a Retrospective Study

**DOI:** 10.3390/brainsci16040374

**Published:** 2026-03-30

**Authors:** Ilaria Guarracino, Marta Maieron, Serena D’Agostini, Miran Skrap, Paola Cogo, Tamara Ius, Barbara Tomasino

**Affiliations:** 1 Scientific Institute, IRCCS E. Medea, Dipartimento/Unità Operativa Pasian di Prato, 33037 Pasian di Prato, Italy; ilaria.guarracino@lanostrafamiglia.it; 2 Department of Physics, University Hospital of Udine, 33100 Udine, Italy; marta.maieron@asufc.sanita.fvg.it; 3 Neuroradiology Unit, Department of Diagnostic Imaging, University Hospital of Udine, 33100 Udine, Italy; dagostini.serena@aoud.sanita.fvg.it; 4 Neurosurgery Unit, Head-Neck and NeuroSciences Department, University Hospital of Udine, 33100 Udine, Italy; miran.skrap@gmail.com; 5 Division of Pediatrics, Department of Maternal and Child Health, University Hospital of Udine, 33100 Udine, Italy; paola.cogo@uniud.it; 6 Academic Neurosurgery, Department of Neuroscience, University of Padova, 35121 Padova, Italy; tamara.ius@gmail.com

**Keywords:** brain surgery, preoperative mapping, task-based fMRI, pediatric population

## Abstract

**Highlights:**

**What are the main findings?**

- The use of fMRI tasks on neurosurgical pediatric population can vary.
- Language and extra-language-related battery fMRI tasks are presented.

**What are the implications of the main findings?**

- Even young patients can reliably perform fMRI tasks.
- The approach proposes a personalized functional assessment of the young patient before surgery.

**Abstract:**

**Background/Objectives**: Performing presurgical functional magnetic resonance imaging (fMRI) mapping in young patients is considered a challenge for clinicians, as fMRI maps are the sole source of information about the functional organization of cognitive functions/areas, especially when an awake craniotomy is not possible, as is often the case for pediatric populations. The literature on the fMRI tasks used in pediatric populations with brain injuries shows a certain heterogeneity in the approaches (task-based or resting states) and tasks, with a preference for motor/language mapping: tasks assessing extra-language functions are lacking. **Methods**: We have designed fMRI tasks focused on language and extra-language functions, which can be easily be applied when clinicians need to perform presurgical mapping. We present a retrospective case series of 17 patients. **Results**: Seventeen young patients (13.4 ± 2.8 years; range 7–16) were included in the study, for whom fMRI was performed. All underwent successful fMRI mapping by completing fMRI tasks selected based on their lesion site. The number of tasks performed by each patient significantly correlated with their age (r(17) = 0.561, *p* = 0.019). The patients tolerated the assessment and had good motion control: their movement parameters were minimal (range of rotation of −0.015–0.01 degrees; range of translation of −0.8–0.2 mm). The most administered fMRI tasks were tongue motor localizer (60%) and object naming (70%), with some patients performing extra-language function mapping involving visuo-spatial processing, selective attention, memory, and inhibition. **Conclusions**: This is an exploratory study given the sample size. fMRI measurements were considered feasible, as patients were able to complete the tasks under clinically realistic conditions. We discuss the clinical implication/usefulness of administering tasks for a personalized functional assessment of the young patient before surgery.

## 1. Introduction

The use of functional magnetic resonance imaging (fMRI) in pediatric patients is becoming increasingly widespread [1]. The most common clinical use of fMRI in a neurosurgical context is for presurgical planning. Clinicians use the fMRI maps to assess the risk of functional deficits after surgery, as these maps highlight functional areas around or in the lesion [2]. In adults, tasks such as finger tapping, and limb or tongue movement are the commonly used tasks for mapping the motor areas, while verb generation, sentence completion, semantic decision, and story processing are used for language mapping [3].

For pediatric populations, it is more difficult to find clear indication in the literature regarding the tasks that can be administered during fMRI scanning. Using fMRI with pediatric patients is always challenging for clinicians. Pediatric patients may not cooperate fully, may lack motivation, and may fall asleep or become tired, which can make the measurements unreliable. They may also be prone to showing agitation and anxiety, resulting in little attention being paid to the task. It is therefore crucial that the fMRI session is suitable for pediatric populations (with suitable images, and an appropriate choice of words, tasks, and cognitive requests). Table 1 provides some examples of the fMRI tasks used with pediatric populations, as reported in the literature [4,5,6,7,8,9,10,11,12,13,14]. Across the studies, different approaches (task-based or resting states) and paradigms emerge; tasks assessing extra-language functions are lacking.

The aim of this study is to compile a list of fMRI tasks focused on both language and extra-language functions than can easily be used to perform a personalized presurgical mapping in the pediatric population (BAMBI-fMRI—Brain Activity Mapping Before Intervention). We tested their usefulness, and we show the feasibility by reporting patients’ motion parameters, the task distribution, and the experience on a consecutive series of pediatric patients who performed the proposed tasks.

## 2. Materials and Methods

### 2.1. Participants

A consecutive series of young patients (age ≤ 18 years) with different etiology is retrospectively reviewed. This study was conducted and reported in accordance with the Strengthening the Reporting of Observational Studies in Epidemiology (STROBE) guidelines for retrospective case series. Exclusion criteria included (i) patients with a history of psychiatric disease or drug abuse, and (ii) developmental language problems or learning disabilities or with a family history for such disabilities; (iii) previous surgery, chemo- or radio-therapy; (iv) precedent biopsy; and precedent surgery for brain glioma.

Inclusion criteria were (i) admission to a neurosurgical ward between 2009 and 2025; (ii) preoperative fMRI assessment; (iii) being native Italian speakers; (iv) having performed fMRI; and (v) having normal or corrected-to-normal vision.

Patients were selected consecutively during the reported period. We did not include etiology as an inclusion criterion because our fMRI protocol was used in all of these cases for presurgical mapping, and we report them here without considering etiology precisely to demonstrate that the fMRI protocol is applicable to all of these cases regardless of etiology, and also because we did not perform subsequent group analyses, where, in fact, etiology could have affected the results of the analyses.

The study was approved by the Ethics Committee (0004890/P/GEN/ARCS, ID 4202).

Parents of each patient provided informed consent to fMRI mapping and surgery, and the procedures were carried out in accordance with the standards of the local ethics committee and the 2013 Fortaleza version of the Helsinki Declaration and subsequent amendments.

### 2.2. MRI Acquisition

In the fMRI scanner, participants laid supine, with their head fixated by firm foam pads. Images were acquired using a 3T Achieva MR whole-body scanner (Philips, Amsterdam, The Netherlands) with a standard 8-channel head coil. High-resolution anatomical images were acquired using a 3D T1-weighted Turbo-Gradient Echo sequence (TR, 8.388 ms; TE, 3.85 ms; voxel size, 1 mm × 1 mm; thickness, 1 mm; number of slices, 190; field of view, 240 mm × 190 mm × 240 mm; acquisition matrix, 240 × 240; flip angle, 8°).

Functional images were obtained using a T2*-weighted Gradient-Echo Echo-Planar Imaging EPI sequence (see Table 2 for acquisition parameters). Slices were acquired in the axial plane, parallel to the anterior commissure/posterior commissure line. The total scanning time depended on the combination of tasks used (see Table 3); we always expect a measurement time of about 60 min (fMRI task plus the anatomical T1 acquisition included the possibility to repeat a task). For example, 13:30 min measurement time for naming, word listening, tongue localizer, and memory fMRI tasks; and 19:54 min measurement time for arrows, visual search, and hand and feet localizers fMRI tasks). Between one task and the following, instructions are repeated to assure that the patient understands the task and few pauses are made.

### 2.3. fMRI Tasks

**Figure 1:**
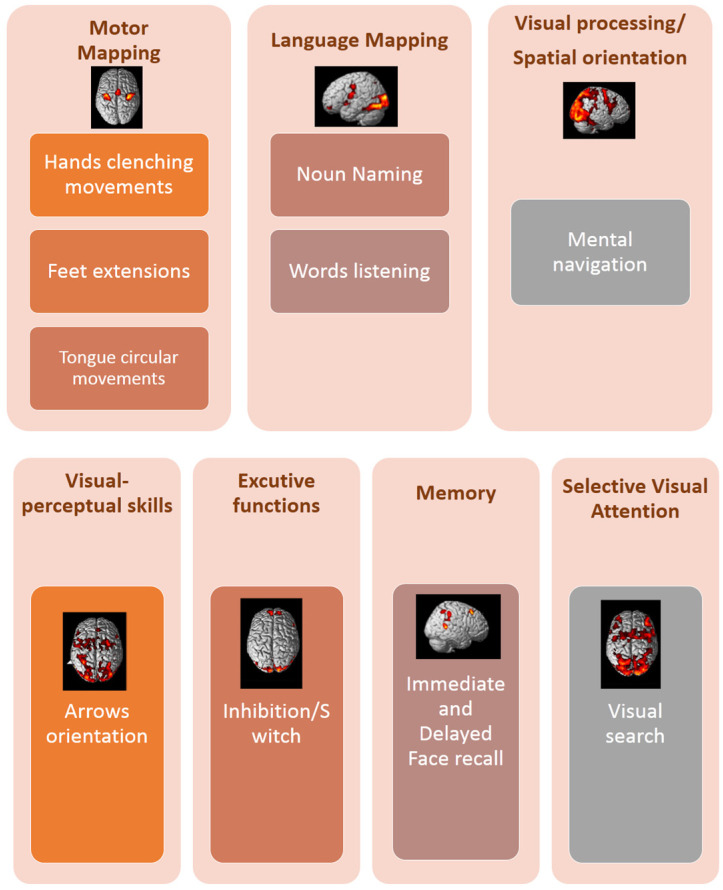
The fMRI battery for performing motor mapping, language mapping, and extra-language-functions mapping.

The fMRI battery included tasks used for performing motor mapping, language mapping, and extra-language function mapping (see Figure 1). A set of these tasks was selected for each patient from those listed in Figure 1/Table 3. No patient performed all of the tasks listed in Table 3. Task selection for individual patients was done according to lesion localization. For example, patients with lesions involving the language network performed the naming, word-listening, and tongue-localizer tasks; patients with lesions selectively involving the hippocampal area performed the mental-navigation and naming tasks. Patients with lesions to the parietal cortex performed the motor tasks, the arrows task, and visual-search task.

Active blocks were alternated with rest conditions, plus two additional resting blocks (one at the beginning of the run and the other at the end). In the rest condition, a fixation cross was presented between blocks; patients were asked to relax (see details in Table 3). Stimuli (see details in Table 3) were programmed and presented using the Presentation software (“Presentation”, Version 9.9, Neurobehavioral Systems Inc., Berkeley, CA, USA) via a VisuaStim Goggles system (Resonance Technology, Northridge, CA, USA). Prior to acquisitions, subjects practiced the tasks outside the scanner.

### 2.4. fMRI Analysis

All calculations were performed on UNIX workstations (SUN Microsystems Computers, Santa Clara, CA, USA) using FSL (www.fmrib.ox.ac.uk/fsl, accessed in 1 Decembre 2025). Data were skull-stripped with BET, motion-corrected with MCFLIRT14, smoothed with gaussian kernel (3 mm FWHM), and registered with FLIRT to the corresponding high-resolution T1 image [13,14,19,20]. The task timing was convolved with the standard double-gamma variate function implemented in FSL, and the fMRI signal was then linearly modeled [20,21] on a voxel-by-voxel basis using a general linear model approach with local autocorrelation correction [19,22] to calculate the subject-specific parameter estimates for each event type. The estimated translation and rotation parameters were added as confounds in the model. At the single-subject level, specific effects were tested by applying linear contrast to the parameter estimates for each event (active vs. rest), and the calculated Z statistic images were thresholded at the whole-brain level using clusters determined with Z > 2.3 voxelwise thresholding and a family-wise error-corrected cluster significance threshold of *p* = 0.05 [21,23].

## 3. Results

### 3.1. Case Series

The patients (nine female and eight male) had a mean age of 13.41 years (SD = 2.87 years, min 7/max 16 years) and a mean level of 7.71 years (SD = 2.87 years, min 1/max 10 years). Preoperative MRI scans revealed lesions in the right (n = 8) and left (n = 9) hemispheres, involving the frontal (n = 4), temporal (n = 4), parietal (n = 5, either pure or involving the temporal or frontal area) cortices, and the hippocampus (n = 2) and occipital lobe (n = 2), with various histologies (see Table 4 for details). All of the patients were right-handed. Many of the assessments we used are routinely carried out before surgery on pediatric populations, including cognitive and psychological assessments. Here, we focus specifically on the functional assessment of presurgical mapping.

### 3.2. fMRI Mapping

We first addressed some methodological details. Patients performed the tasks successfully. fMRI maps were considered feasible, as patients completed the tasks. In detail, our approach is not to have every patient perform every task, but rather to select tasks based on the location of their lesions. Therefore, simply counting the number of patients who performed each task is misleading. We present the number of patients who performed the tasks, stratified by area and function in Table 5.

**Figure 2:**
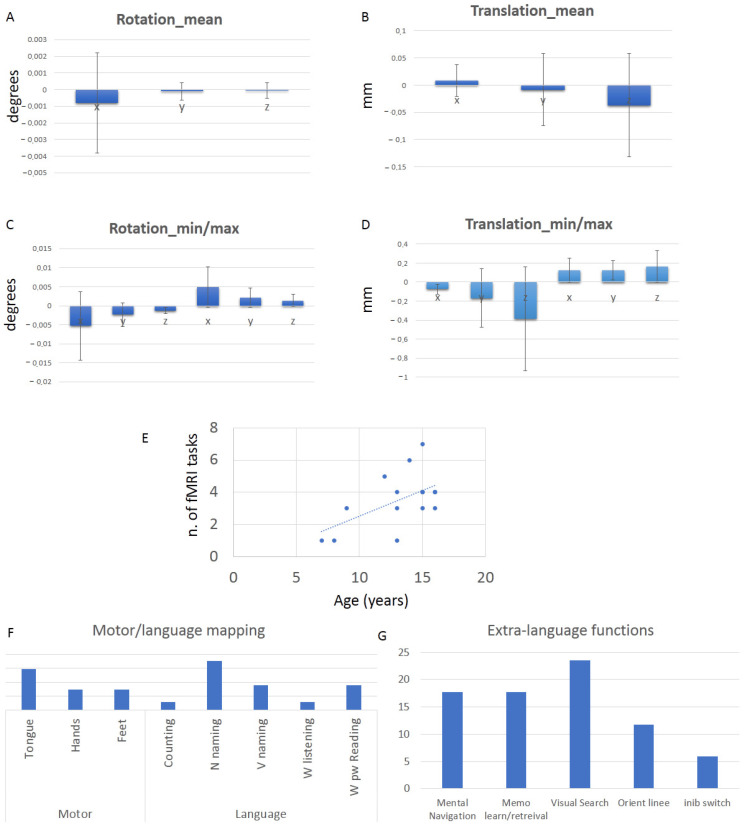
Results from fMRI scanning. Motion-related parameters: rotation mean (**A**) and range (**C**), translation mean (**B**) and range (**D**); correlation between the number of tasks performed and age (**E**); percentage of tasks performed in language-related (**F**) and extra-language (**G**) domains.

Their movement parameters resulted well within the 3 mm/3 degrees rule (see Figure 2). In detail, we report the mean rotation on the x (0.0008 ± 0.003), y (−0.0002 ± 0.0005), and z (−0.00005 ± 0.0004) planes, as well as the mean translation on the x (0.009 ± 0.029), y (−0.0023 ± 0.0030), and z (−0.04 ± 0.095) planes. We report the rotation range on the x (−0.0052 ± 0.009 min; 0.00498 ± 0.0053 max), y (−0.0002 ± 0.0005 min; 0.002077 ± 0.00255 max), and z (−0.0012761 ± 0.000799 min; 0.001 ± 0.002 max) planes, as well as the mean translation on the x (−0.07 ± 0.053 min; 0.124 ± 0.128 max), y (−0.17 ± 0.309 min; 0.124 ± 0.102 max), and z (−0.39 ± 0.545 min; 0.163 ± 0.166 max) planes.

Not surprisingly, the number of tasks performed by each subject significantly correlated with patients’ age (r(17) = 0.561, *p* = 0.019). Tongue motor localizer and object naming were the most administered fMRI tasks (see Figure 2).

#### 3.2.1. Relationship of fMRI Map with Intraoperative Stimulation Mapping

Table 6 shows that, of the 17 patients, six had an fMRI cluster located close to the border of the lesion; of these six, four patients underwent awake surgery (one patient had a lesion that was too large for this procedure, and the other patient underwent motor stimulation mapping under general anesthesia). Of the five patients who underwent cortical stimulation mapping, four had a positive stimulation site (two had a language-related positive site and two had a motor-related positive site). Resection was total for all of them, as the positive sites were always found at the margin of the lesion. However, for one patient, resection was stopped when a positive site was found within the lesion. There are too few data points to perform a statistical analysis. The remaining 11/17 patients were operated on under general anesthesia, with neuronavigation performed before, during, and at the end of resection. On the T1/T2 MRI scans used for neuronavigation, the fMRI maps were overlaid so that the surgeon could check the position and resection borders throughout the procedure

#### 3.2.2. Case Series Examples

We next present how the selected tasks may add important information for presurgical planning. As mentioned before, the selection of the fMRI tasks is a personalized approach based on the patient’s age, her/his status/collaboration, and the lesion site. Data are presented at individual examples since we did not perform second-level group analyses (due to all the sample heterogeneity, i.e., different pathologies, different lesion site, and different selection of test per lesion site).

##### Visual Processing/Mental Navigation, Language Mapping, and Motor Localizers

**Figure 3:**
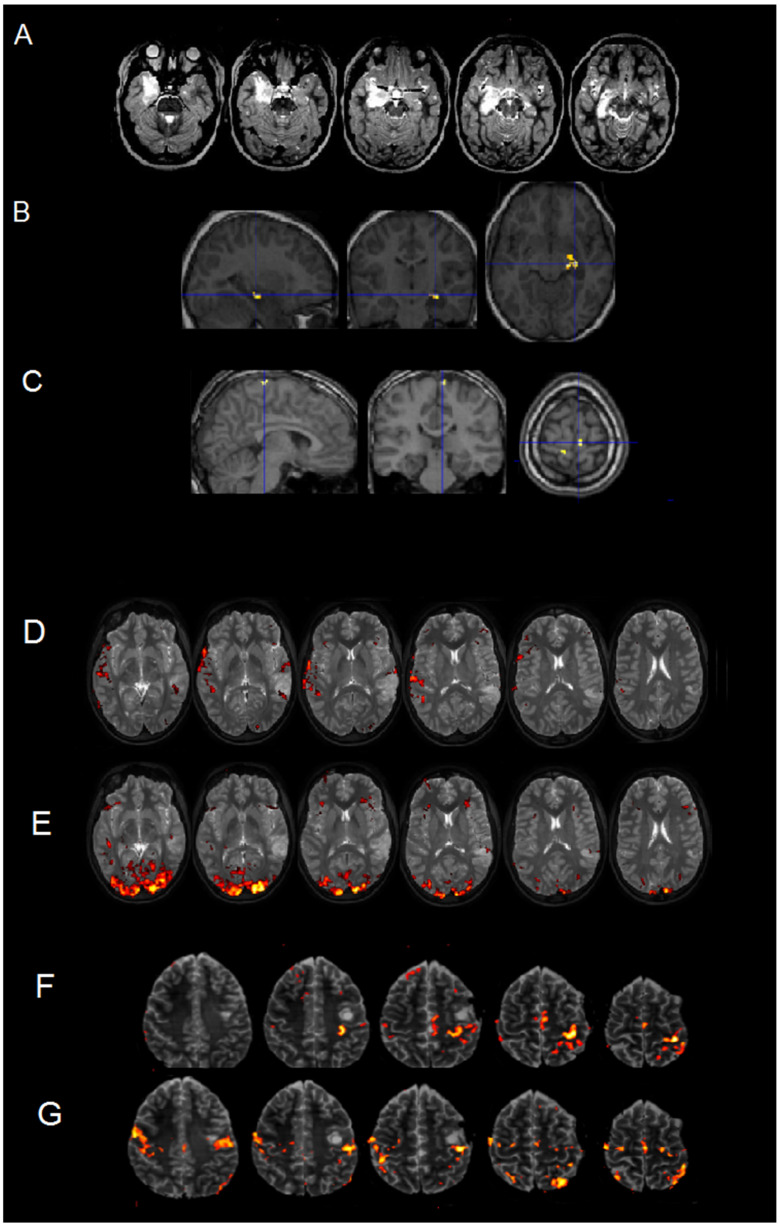
Mental navigation task-related activations in a patient with a right hippocampal lesion (**A**). The task activated the left, contralesional, hippocampus, (**B**) but not the right, contralesional, hippocampus. In addition, the mental imagery showed that the feet area of the primary motor cortex was also activated (**C**). Language mapping was performed in a patient with a left posterior temporal lesion. Object naming did not produce a clear map (**E**), whereas a passive word-listening task did (**D**), showing activation related to language processing in/around the lesion. In a second patient, the hand area representation was localized just posteriorly (**F**), while a motor tongue localizer evidenced activation related to tongue movements in/around the lesion (**G**).

With young children (like an 8-year-old boy, P#10), only one fMRI task was used, as the lesion involved the right hippocampus. A mental navigation task was used as a localizer for the hippocampus (see Figure 3A,B). By using a mental imagery task asking the patient to imagine walking in a labyrinth, the feet area of the primary motor cortex is also activated (see Figure 3C).

As for language mapping, for example, in a 15-year-old boy (P#2) with a left temporal lesion, a word-listening task (see Figure 3D) was administered. An object-naming task elicited poor activation (Figure 3E), while the listening task succeeded in order to obtain activations in the expected areas, as the task requires passive listening, and thus minimal collaboration. In this patient, the task showed that the ipsilesional auditory cortex is not activated likely because the area is invaded by the lesion.

Motor mapping was performed on a 16-year-old girl (P#3) with lesions close to the motor areas (see Figure 3F). The hand localizer activated a cluster posterior to the lesion (Figure 3G), while the tongue localizer activated a cluster in the motor representation posterior to the lesion with premotor activation right next to the lesion (Figure 3F).

##### Extra-Language Functions

The fMRI tasks also aim at mapping extra-language functions in order to include tasks suitable for mapping those areas not directly involved in language processing or motor control.

**Figure 4:**
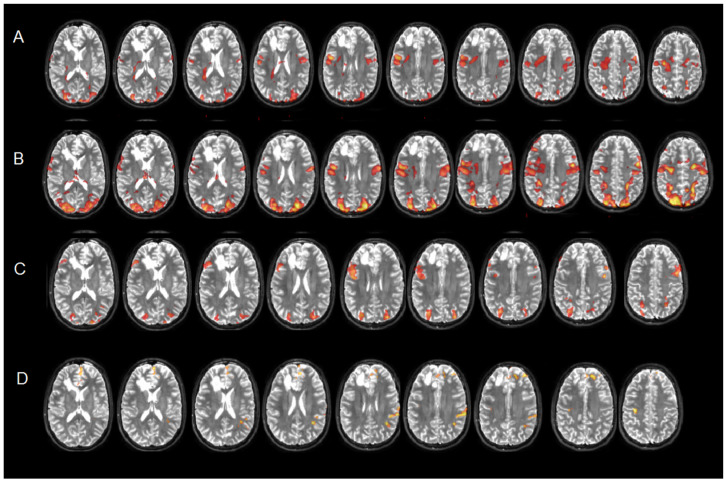
Extra-language mapping in a patient with a lesion in the right prefrontal cortex, involving a visual/perceptual (**A**) visual selective attention (**B**) and memory (with the contrast recall vs. learning (**C**) and learning vs. recall (**D**)) tasks.

For example, patients with frontal or parietal lesions were given tasks to test their visual/perceptual skills and visual selective attention. For a patient with a lesion in the right prefrontal cortex (Figure 4A), we administered a visual/perceptual task that activated a bilateral frontal cluster posterior to the lesion (Figure 4A), as well as a task that tested visual selective attention and showed a cluster of activation just above the lesion (Figure 4B). The contrast between recall and learning showed activation of a cluster lateral to and on the border of the lesion (Figure 4C), while the contrast between learning and recall showed activation of a cluster behind and on the border of the lesion (Figure 4D).

## 4. Discussion

CNS tumors diagnosed in young patients and adolescents pose a complex surgical challenge due to their possible proximity to functional areas and the delicate nature of the functions involved in an individual’s development. Among the methods used to obtain functional information, fMRI has the advantages of being noninvasive, of being able to be performed prior to surgery to guide surgical decisions, and of allowing mapping of brain areas involved in cognitive functions [22,24].

One of the methodological issues when performing fMRI scans on a pediatric population is the amount of head movement (e.g., translations and rotations), which can interfere with image quality. The authors [15] studied children’s head movements during fMRI tasks and found that head movement is a stable individual’s biological characteristic influenced by cognitive and behavioral factors. According to the authors, neuroimaging studies of the pediatric population must therefore consider these aspects to avoid misinterpreting the data [15,23]. Others [24,25] have found differences in the amount of head movement based on gender, age, and the type of fMRI task performed. In our sample, we observed stable and limited (comparable to adults) movement parameters, supporting the idea that fMRI is feasible in young patients and can be used for preoperative planning.

Using young patient-friendly MRI tasks is an important aspect. In our experience, both the preoperative assessment and the tasks used in fMRI belong to neuropsychological batteries created specifically for the pediatric population, e.g., NEPSY [15]. This methodological choice enabled us to use fMRI stimuli that were highly familiar and salient, simple from a perceptual point of view, and belonging to semantic categories specific to young patients, as also indicated by other works [4,6,25,26]. This generates interest in the young patients, who do not become bored, as could happen if they were faced with tasks that were too complex. In our sample, the young patients were able to complete the tasks without the need for interruption, repetition of instructions, or misinterpretation of the task. The number of tasks varied according to the patients’ ages, as is highly understandable. In addition, using specific tasks for each lesion site, as in our sample, meant that the young patient only had to spend a short time in the scanner, which optimized performance and limited the effects of fatigue. This enabled us to conduct shorter sessions, thus avoiding the need to divide fMRI acquisition in multiple runs performed at different times throughout the day, as recommended by some authors [16,27]. Finally, it is also important to motivate young patients to understand the importance of the fMRI measurements. In this case, the neuropsychologist plays a key role in engaging both the patient and their caregivers.

Overall, although our experience of fMRI scanning on a pediatric population is limited, the fMRI maps have always been informative in terms of presurgical planning. Analysis of individual cases revealed that information about the functional pattern of areas in/around the lesion was obtained. This is often the only information the team can obtain about the functional status of the areas involved in the surgery, since awake surgery is rarely performed on such young patients. A proposal for the future is to create more selective tasks activating the brain networks supporting cognitive processing.

This preliminary experience is based on a small sample size (17 patients), a very heterogeneous group in terms of pathologies, with different lesion sites. All of these factors may influence the results and limit the generalizability of the study. The lesion site itself may have influenced the observed activation patterns so that reference methods in the context of pediatric neurosurgery, such as direct cortical stimulation (when feasible of young population) or postoperative functional outcome, remain the gold standard.

### Limitation of the Study

A primary limitation is the absence of some form of validation against a clinical reference standard, such as direct cortical stimulation, intraoperative mapping, postoperative functional outcomes, or established noninvasive comparators (for example, MEG or TMS). Future work will involve collecting and analyzing these data in order to report quantitative concordance in a quantitative manner, such as the spatial relationship between fMRI activation peaks and stimulation-positive sites; agreement in hemispheric dominance for language; or the extent to which mapped regions predicted postoperative deficits or informed surgical boundaries.

We acknowledge that our sample size is limited, which prevented us from performing second-level group analyses that may also reveal plasticity-related changes. In addition, the case series comprises a heterogeneous group of pathologies. The aim of this study was to present a series of tests for use in fMRI with pediatric populations with stimuli suitable for subjects of this age and engaging and feasible tasks. This limitation did not allow us to perform second-level group analyses that could also reveal tumor-type-related effects on networks activation. Secondly, we did not include a control sample of healthy young patients. This aspect may have limited the comparisons we made. Future studies may include a control group for comparison with the performance of the pediatric clinical population. A more systematic exploration would have included results from areas activated by the different tasks in healthy individuals of the same age as the patients.

Lastly, we acknowledge that the exclusion criteria used in this study removed several groups that are common in pediatric neurosurgical practice. These groups include young patients with developmental language or learning difficulties and those who have undergone prior interventions. This likely enriches the sample with high-functioning and cooperative patients, which may therefore overestimate the feasibility of the procedure in an unselected presurgical population. Conversely, even when working with adults (not to mention young patients), it is recommended that fMRI is performed on patients who can perform the tasks [17,26].

## 5. Conclusions

Our experience is preliminary, as our results are based on a small and heterogeneous sample. Nonetheless, in our small experience, fMRI on pediatric neurosurgical population was possible and provided important information prior to surgery thanks to the cooperation of young patients. Undoubtedly, the proposed protocol of fMRI tasks requires further validation in prospective studies with larger sample sizes. Only further evidence could test its potential clinical impact.

## Figures and Tables

**Table 1:** Example of the fMRI tasks used in in pediatric population with brain injuries.

Reference	fMRI Task	n. Patients/Pathology	Conclusion
Charbonnier L. et al. (2020) [4]	Story listening, letter–picture matching, verb generation	32 healthy children (6–10 yrs) + 7 with epilepsy (7–11 yrs)	Designing tasks that match children’s interests and abilities allows for reliable mapping of language areas in both healthy children and those with epilepsy. Child-friendly tasks enable assessments of laterality.
Desai V.R. et al. (2019) [5]	rs-fMRI vs. tb-fMRI (verb generation, sentence completion)	29 children with epilepsy	Very high rs/tb-fMRI concordance (0.93); rs useful if uncooperative.
Genetti M. et al. (2013) [6]	Auditory semantic decision (8 min)	28 healthy controls and 35 consecutive patients with focal epilepsy or a brain tumor.	The proposed language tb-fMRI techniques have reliably localized language areas, becoming a valuable additional tool for surgical planning for seizure foci and brain tumors.
Hoang D.H. et al. (2019) [7]	Working memory (block-design)	23 controls + 11 post-medulloblastoma (8–14 yrs)	The use of rs-fMRI and tb-fMRI can improve surgical outcomes.
Krafft H. and Staudt M. (2022) [8]	Vowel identification task, word-chain task, beep-story task, synonym task	114 pediatric cases (5.8–17.8 yrs) with different etiologies	Based on 13 regions of interest (ROIs) valid for language lateralization (VLR_old), it was possible to classify language dominance. Four new task-specific VLRs (VLR_new) were identified, consistent with language dominance in patients. An optimal order for the q tasks was proposed.
Shurtleff H.A. et al. (2022) [9]	Memory autobiographical task encoding + retrieval (inside/outside scanner); language protocols involved verb and sentence generation	72 patients (6.7–20.9 yrs) with seizure focus and/or tumor	Bilateral activation of temporal lobe. A clinically applicable functional MRI memory paradigm for use with pediatric patients.
Jacola et al. (2006) [10]	Verb generation + story listening	3 children with perinatal stroke + 39 controls	In stroke pt, activity during verb generation tends to be bilateral or shifted towards the right hemisphere, while in controls, activation is strongly lateralized to the left. In subjects with lesions, there is bilateral or right-sided activation in story-listening tasks. Reorganization of language after stroke.
Sarlo et al. (2025) [11]	Paragraph-reading task	41 children with epilepsy (6–16 yrs)	There was no significant difference in fMRI activation during the paragraph-reading task between the groups. Better reading skills correlated with greater activation in the left superior temporal gyrus. Struggling readers show less activation in specific areas: bilateral temporal gyri, left cerebellum, and right inferior frontal gyrus. This paradigm is robust for identifying the language network, for example, for presurgical language mapping in children with epilepsy.
Mankinen et al. (2015) [12]	Reading, story listening, memory encoding, memory retrieval	21 children with epilepsy + 21 control	Different BOLD activation only in story listening (thalamus right, basal ganglia right). In patients with TLE, activation was increased compared to controls, and deactivation was decreased in the story-listening task.

**Table 2:** MRI acquisition parameters.

Task	TR (ms)	TE (ms)	Voxel Size (mm)	Thickness (mm)	Number of Slices	Field of View (mm)	Acquisition Matrix	Flip Angle	Number of Volumes	Number Task_Blocks/Rest_Blocks	Duration Task_Blocks/Rest_Blocks	Total Time (min:s)
Arrows	2500	35	1.797 × 1.797	3	29	230 × 88.33 × 230	128 × 128	90	132	9/10	20 s/15 s	5:30
Visual search	2500	35	1.797 × 1.797	3	29	230 × 88.33 × 230	128 × 128	90	118	8/9	20 s/15 s	4:54
Memory	2500	35	1.797 × 1.797	3	29	230 × 88.33 × 230	128 × 128	90	126	8/9	22.5 s/15 s	5:15
Inhibition/switch	2500	35	1.797 × 1.797	3	29	230 × 88.33 × 230	128 × 128	90	186	15/16	15 s/15 s	7:45
Naming	2500	35	1.797 × 1.797	3	29	230 × 88.33 × 230	128 × 128	90	78	6/7	15 s/15 s	3:15
Word listening	2500	35	1.797 × 1.797	3	29	230 × 88.33 × 230	128 × 128	90	66	5/6	15 s/15 s	2:45
Hands localizer	2500	35	1.797 × 1.797	3	29	230 × 88.33 × 230	128 × 128	90	102	8/9	15 s/15 s	4:15
Feet localizer	2500	35	1.797 × 1.797	3	29	230 × 88.33 × 230	128 × 128	90	102	8/9	15 s/15 s	4:15
Tongue localizer	2500	35	1.797 × 1.797	3	29	230 × 88.33 × 230	128 × 128	90	54	4/4	15 s/15 s	2:15
Mental navigation	2500	35	1.797 × 1.797	3	29	230 × 88.33 × 230	128 × 128	90	108	8/8	15 s/15 s	4:30

**Table 3:** fMRI battery.

Task	Function Examined	Task Description	nr Stimuli per Block/Total nr Stimuli/Each Stimulus Lasts (Seconds)
Arrows [15]	Visual perceptual skills	The child sees a target and a variable number of arrows, which are marked with a number that may or may not point in the target’s direction. They have to say which of them, point to the target.	5/50/4
Inhibition/switch [15]	Ability to inhibit automatic responses in favor of new ones	The task consists of three parts. The stimuli are white or black squares and circles distributed randomly. 1—Say the name of the shapes one sees (squares and circles). 2—Say the opposite name of the shape one sees: if it is a square, say circle. 3—When the shape is black, one says the correct name of the shape; when the shape is white, one has to say the opposite name.	1/15/15
Memory [15]	Decoding facial features; recognizing and distinguishing faces; memory and learning non-verbal material	The task consists of two parts 1—Black-and-white faces are shown, and the subject must press key 1 if it is a male and key 2 if it is a female. 2—Triplets of faces are shown, and the subject must press keys 1 to 3 to indicate which face they saw previously.	4/15 learning + 15 recognition/5
Visual search [15]	Selective visual attention	The subject is presented with a target face (a girl). The task is to recognize all faces that are the same as the target during the scan by pressing a button (right hand) that corresponds to “yes” and a button (left hand) that corresponds to “no”. The second scan has a boy’s face as the target. The drawings are in black and white.	20/160/1
Naming [16,17]	Naming ability, retrieval of the correct lemma	The subject is asked to covertly name a series of black-and-white images depicting objects/animals.	8/46/1.875
Word listening [18]	Auditory processing; word listening	The subject is asked to pay attention to the words.	12/216/1.25
Motor tongue movement	Tongue circular movements	Subjects are asked to move their tongue inside their mouth, which must be kept closed. The “task” moments alternate with pause moments in which the subject must remain still and do nothing.	4/4/15
Motor hands movement	Hands clenching movements	With their hands at their sides and palms facing upwards, subjects are asked during the “task” phases to open and close their hands until the word “move” appears on the screen. The task phases alternate with “rest” phases in which they do not have to do anything.	8/8/15
Motor feet movement	Feet extension movements	Subjects are asked to move their foot by pointing their toes up/down every time the word “move” appears on the screen. During the “rest” phases, they must do nothing and remain still.	8/8/15
Mental navigation	Perceptual/mental navigation	The subject is presented with videos showing a labyrinth room with the camera moving inside. They imagine walking in and exploring the space. During the “rest” phases, they must do nothing and remain still.	3/24/5

**Table 4:** Patients’ clinical and demographic details.

Case	Age	Education	Sex	Histology	Site	Side
1	16	10	m	Pilocytic astrocytoma/pleomorphic xanthoastrocytoma	Frontal	RH
2	15	9	m	Oligodendroglioma	Temporo-parietal	LH
3	16	10	f	Papillary glioneuronal	Frontal	LH
4	13	7	m	Pilocytic	Fronto-parietal	LH
5	13	7	f	Glioneuronal tumor	Occipital	LH
6	7	1	m	Glioneuronal tumor	Occipital	RH
7	15	9	f	Glioneuronal tumor	Temporal	LH
8	12	6	m	DNET	Temporo-parietal	LH
9	16	10	m	Low-grade I	Frontal	RH
10	8	2	m	Glial tumor	hippocampus	RH
11	9	3	f	Glioblastoma	Parietal	RH
12	13	7	f	Expansive lesion ganglioglioma	Temporal	RH
13	16	10	f	Dysplasia	Frontal	RH
14	15	9	m	Cavernoma	Temporal	LH
15	15	9	f	Arteriovenous malformation	Temporal	LH
16	14	8	f	Cavernoma	Parietal	RH
17	15	9	f	Sclerosis	Hippocampus	LH

**Table 5:** Summary of the tasks performed by the patients.

Site	Motor Localizers	Language Localizers	Mental Navigation	Extra-Language Tasks
Hippocampus/occipital cortex			4/4	
Frontal or fronto/parietal	4/5	4/5		3/5
Temporal or temporo/parietal	5/6	6/6		2/6
Parietal	2/2	2/2		

**Table 6:** fMRI mapping results and intraoperative technique, along with direct cortical stimulation-mapping details.

Case	fMRI Cluster Close to the Lesion (1)/Far from It (0)	fMRI Cluster Detail	Awake Surgery (1)/General Anesthesia (0)	Stimulation/Mapping Positive (1)/Negative (0)	Stimulation Details	Extent of Resection
1	1	Naming-related act. lateral/close	1	0	Mapping by language and subcortical areas. Negative response.	total
2	1	Naming-related act. anterior and within	1	1	Cortical monitoring plus tests. Partial removal of the neoplasm, constant intraoperative subcortical monitoring. Positive response for language (naming)	partial
3	1	The motor part of speech: superior, lateral and contiguous	1	1	Negative language stimulation, removal of the lesion clearly defined with respect to the parenchyma. In the final part, fluency worsens; stimulation causes speech arrest. However, this part appears healthy close to the lesion.	total
4	1	Hands: posterior, lateral, contiguous	1	1	Lesion in front of the motor strip and mapping shows motor response.	total
5	0		0	-	-	total
6	0		0	-	-	total
7	0		0	-	-	total
8	0		0	-	-	total
9	0		0	-	-	total
10	0		0	-		total
11	1	Hands: superior, close	0	1	Mapping only for arm above the right margin of the lesion	total
12	0		0	-	-	total
13	1	Naming: anterior, contiguous	0	-	-	total
14	0		0	-	-	total
15	0		0	-	-	total
16	0		0	-	-	total
17	0		0	-	-	total

## Data Availability

The materials that support the findings of this study are available from the corresponding author upon reasonable request due to privacy.

## Abbreviations

The following abbreviations are used in this manuscript:

BAMBI	Brain Activity Mapping Before Intervention Tasks
fMRI	functional magnetic resonance imaging
